# Workplace Loneliness: The Benefits and Detriments of Working From Home

**DOI:** 10.3389/fpubh.2022.903975

**Published:** 2022-05-27

**Authors:** Amy Wax, Caleb Deutsch, Chloe Lindner, Steven J. Lindner, Andrea Hopmeyer

**Affiliations:** ^1^Department of Psychology, California State University, Long Beach, CA, United States; ^2^Department of Psychology, Occidental College, Los Angeles, CA, United States; ^3^Department of Psychology, Fordham University, New York City, NY, United States; ^4^The WorkPlace Group, Florham Park, NJ, United States

**Keywords:** workplace loneliness, working from home (WFH), affective organizational commitment, coworker support, supervisor support, OCBs, perceived performance

## Abstract

Self-determination theory posits that relatedness and autonomy are two drivers of work-relevant outcomes. Through the lens of this theory, the current study explored the potential interactive effects of relatedness and autonomy on affective, relational, and behavioral outcomes at work, operationalizing relatedness as workplace loneliness and autonomy as the ability to work from home. To test this relation, survey-based data from a sample of 391 working adults were collected and a path analysis was carried out. Results suggested that workplace loneliness negatively predicts affective organizational commitment, perceptions of coworker and supervisor support, organizational citizenship behaviors, and perceived performance. Furthermore, results suggested that workplace loneliness and working from home have an interactive effect on affective organizational commitment, perceptions of coworker support, and organizational citizenship behaviors. Specifically, working from home had a beneficial impact on the relation between workplace loneliness and affective organizational commitment/perceptions of coworker support, but a detrimental impact on the relation between workplace loneliness and organizational citizenship behaviors. These results add to the extant body of scholarly work of Self-Determination Theory by testing the theory in the post-pandemic context of working from home. In addition, these results have practical implications for managers, who should strive to create opportunities for employees who work from home to enact organizational citizenship behaviors.

## Workplace Loneliness: The Benefits and Detriments of Working From Home

Due to the global COVID-19 pandemic, estimates of the number of U.S. employees ***working from home***—either part- or full-time—have ranged from 58% (1) to 71% (2). These figures suggest that the proclivity of American workers to work from home has *at least* doubled, and has possibly nearly quadrupled, since prior to the onset of the pandemic (2, 3). This rapid and drastic change in worker demographics poses a number of questions about the efficacy and implications of working from home.

Prior literature has suggested a number of potential detriments to working from home. For instance, research has indicated that individuals who work from home experience increased levels of stress, irritability, worry, and feelings of guilt when compared to other workers (4). Other scholarly evidence has suggested that working from home reduces happiness (5). However, there have also been positive implications of working from home in the scholarly literature. For example, demographic research by the Pew Research Foundation has shown that over half of individuals would prefer to continue working from home after the pandemic has concluded (2), suggesting that there may be perceived benefits to working from home. Some research has even suggested that working from home may lead to performance increases (6).

The COVID-19 pandemic has also impacted experiences of loneliness. Recent research has suggested that the pandemic has significantly increased self-reported loneliness, particularly among people who were under orders to stay at home (7). In particular, ***workplace loneliness***—or, the feeling that one's social needs are not being met at work (8)—is important to consider in the context of working from home. Traditionally, working from home has been shown to increase feelings of loneliness (4), although this finding has yet to be replicated during the course of the COVID-19 pandemic, as far as the authors are aware. Workplace loneliness has also been shown to have a detrimental impact on critical, work-relevant outcomes such as performance (8).

Both workplace loneliness and working from home can be viewed *via* the lens of ***self-determination theory*** [SDT; (9)], which states that individuals are motivated at work by relatedness and autonomy. Relatedness, or the need to feel connected to others, is a facet of SDT that workplace loneliness maps on to, whereas autonomy, or the need to feel in control of one's own life, is a facet of SDT that working from home maps on to. Thus, an open question is whether workplace loneliness might *interact* with the experience of working from home. As far as the authors are aware, there is no extant literature on this topic, as of yet. Will working from home mitigate any negative impacts that loneliness has on work-relevant outcomes, or will it exacerbate them?

## Contributions of the Current Study

The current study sought to make three contributions to the literature. First, from prior research we know that SDT (9) applies to human motivation in a number of different contexts [e.g., (10–13)]. However, limited work has been done on SDT in the context of working from home, which is of concern because of the recent spike in working from home due to the COVID-19 pandemic. Accordingly, this paper addresses this concern by examining this novel application of SDT, which will contribute to the literature by extending SDT to an increasingly common modality of work—namely, working from home.

Second, the extant literature has explored the outcomes of general loneliness in substantial depth, but has focused less on the repercussions of workplace loneliness, specifically. This gap in the literature is problematic because, just like general loneliness [e.g., (14)], workplace loneliness has the potential to have a measurable, detrimental impact on individual outcomes. Thus, the current study contributes to the literature by investigating the impact of workplace loneliness on a variety of work-relevant outcomes, clarifying these relationships for both scholars and practitioners to ground future work in.

Finally, the potential interactive effect workplace loneliness and working from home on work-relevant outcomes has yet to be thoroughly investigated in the extant literature. This potential relation is of particular importance to explore given the fact that, as previously stated, working from home is increasingly common in the post-pandemic era. Accordingly, the current paper contributes to the literature by unpacking the potential moderating impact of working from home on the aforementioned relations between workplace loneliness and outcomes, which may help guide future scholarly and applied work.

## Workplace Loneliness

Loneliness is defined as a perceived deficit in one's desired social relations. A common misconception about loneliness is that it occurs due to an insufficient number of personal relationships, when in fact it relates more closely to a dearth of high-quality relationships. Rather than being solely related to the number of relationships a person has, loneliness most closely reflects whether or not one has achieved their desired amount of quality contact, no matter how many or few people that may require (15).

Workplace loneliness is the feeling that one's social needs are not being met at work (8), and is often conceptualized as having two distinct facets: emotional deprivation and social companionship. Emotional deprivation is defined as a failure to emotionally connect with or attach to others, and can lead to a variety of undesirable workplace outcomes such as a drop in organizational citizenship behaviors and performance (16). Social companionship is the connection to and engagement with a network of people (17–19), and has been shown to positively predict affective organizational commitment and negatively predict the intention to seek new employment (20). Social companionship has also been shown to predict both intrinsic and extrinsic forms of job satisfaction (21).

Prior research has indicated that workplace loneliness is impacted by a number of factors. For example, a meta-analysis of the loneliness literature found that the extent of one's desire for social relationships at work predicts loneliness at work. This study also reported that individual factors such as social skills predict loneliness at work, because those with a better ability to communicate and socialize with others have more opportunities to develop high-quality, satisfying relationships (22). In a similar vein, social information processing and social skills have been shown to negatively predict loneliness at work (23). Shyness and introversion also predict loneliness at work, while extraversion serves as a protector against the experience of workplace loneliness (22). Finally, research carried out on prison staff in Turkey revealed that job satisfaction negatively predicts loneliness at work, as well (24).

In terms of work-relevant correlates, loneliness at work has been shown to be positively associated with role conflict—an inability to understand one's role at work—and role ambiguity—not understanding the expectations of one's role at work—and to negatively correlate with opportunities for friendship at work and the perceived quality of workplace friendships (25). Loneliness at work has also been shown to have negative consequences for one's health (26, 27). With regard to mental health, a study of managers across New Delhi found that loneliness at work was negatively associated with psychological well-being and self-esteem. Additionally, managers suffering from loneliness at work reported increased feelings of work alienation (26). Moreover, loneliness appears to have implications for physical well-being. For instance, a study that utilized a sample of over 10,000 individuals across 14 countries found that loneliness predicts developing a work disability (27).

While the literature on specific job types predicting loneliness at work is limited, one type of work that may be associated with increased loneliness at work is temporary work. Compared to their permanent counterparts, temporary workers report higher levels of loneliness at work (28). Furthermore, research on job roles has revealed patterns related to workplace loneliness. Contrary to the truism about it being “lonely at the top,” research has indicated that those in leadership positions (who may not have many peers in the workplace) do not feel any lonelier, on average, than their subordinates with numerous coworkers at the same level (29). Circling back to the definition of workplace loneliness, this finding implies that, although leaders may have less relationships at work, their relationship quality does not suffer.

## Working From Home

During the Oil Crisis of 1973, when the worldwide price of oil rose nearly 300% (30), individuals and organizations alike were forced to quickly curtail their oil consumption. Many businesses responded by softening the requirement to commute to/from work, consequently reducing traffic congestion and energy consumption. Eventually, the term telecommuting^1^—or, the use of “telecommunications technology to partially or completely replace the commute to and from work” [(31), p. 273]—was coined to refer to this practice.

Long after the end of the oil crisis, companies have continued to embrace the practice of working from home, which simultaneously helps to manage work-home relations and satisfy both the Clean Air Act and the ADA requirements (32). Work-from-home arrangements also reduce employer's overhead costs and associated expenses (33); research has shown employers can save about $11,000 a year for every person that works remotely half of the time (34). Telecommuting also has a positive impact on turnover rates, with job attrition rates falling by over 50% (6). Moreover, work-from-home employees appear to work longer hours in order to compensate for time away from work (35).

In contemporary times, with the evolution of technological capabilities and the COVID-19 pandemic, working from home has become a “new normal” (36). For example, Facebook is planning for permanent remote workers and stated that within a decade as many as half of the company's more than 48,000 employees would work from home (37). Other large companies such as Google, Microsoft, and Apple are expected to follow suit (38).

Several studies have noted a positive relationship between productivity and telecommuting (39–43). In line with this finding, IBM has also noted that the productivity rate for telecommuters is 10 to 20 percent higher than their office-based workers (41). Telecommuting has been shown to do more than just help the employer's bottom line, however; it also has a positive impact on employee outcomes. For instance, research has shown that telecommuters experience less stress, less work-life conflicts, higher work engagement, and increased job performance when compared with their counterparts in the office (42, 44). Furthermore, research has indicated that the option to choose when and where to work is positively correlated with work engagement and negatively correlated with exhaustion, potentially because of more effective and efficient electronic communication with co-workers (45).

Contrasting with the benefits of working from home, many studies have observed higher levels of loneliness among telecommuters in comparison to non-telecommuters (4, 46–49). For example, a study by Mann and Holdsworth (4) found that 67% of telecommuters reported loneliness, whereas loneliness was not reported by any of the traditional, non-telecommuting workers who were surveyed. Other work has suggested that individual differences may impact experiences of workplace loneliness; for instance, telecommuting mothers have been shown to report higher levels of loneliness than telecommuting fathers (48).

## The Application of Self-Determination Theory

Self-determination theory [SDT; (9)] states that human beings have three innate categories of psychological need that serve to motivate them: a) competence, or the need to feel capable, b) autonomy, or the need to feel in control of one's life, and c) relatedness, or the need to feel connected to others. Specifically in terms of relatedness needs, research on SDT has shown that a lack of fulfillment may result in a variety of outcomes. For instance, variations in assessments of relatedness throughout the course of the day have been shown to map onto experienced emotions (12, 13). Moreover, research has suggested that relatedness needs are closely associated with actual relationship quality, above and beyond the impact of competence and/or autonomy (10, 12). Finally, perceptions of relatedness have been shown to impact the way that individuals behave, including executing prosocial behaviors (11).

The current study focuses on the impact that a dearth in relatedness has on work-relevant outcomes; loneliness is, by definition, a lack of fulfillment in terms of relatedness, as lonely individuals sense that their current social relationships do not fulfill them adequately. Thus, in order to investigate the potential impacts of workplace loneliness, we chose to map our explored correlates of loneliness onto the preexisting pattern of correlates for SDT relatedness needs. Accordingly, we chose to emphasize the following three workplace experiences: a) affect, b) relationships, and c) behaviors.

### Workplace Loneliness and Individual Affect

Work-relevant affect refers to one's moods and emotions while at the workplace. Affect can be either positive (enthusiastic, alert, excited) or negative (distressed, fearful, nervous) (50). Affect is of importance to employers, as it can have an impact on everyday business practices. For instance, both individuals and groups have been shown to exhibit increased prosocial and helping behaviors when reporting better moods (51). Positive affect at work has also been shown to positively correlate with performance (52). Furthermore, affect has also been found to predict turnover intentions; positive mood acts to protect against turnover whereas negative affect promotes turnover intentions (53).

Affective organizational commitment, as conceptualized by Allen and Meyer (54), is the affective and emotional attachment one has to their organization such that the individual identifies with, is involved in, and enjoys membership in the organization. Affective organizational commitment is a particularly important form of affect to investigate because it is associated with a variety of workplace outcomes. For example, affective commitment is negatively predictive of turnover intent and positively predictive of job performance (55, 56), and is also positively associated with organizational citizenship behaviors (57).

From the lens of SDT, a lack of relatedness has been shown to be related to emotional processes at the individual level. For example, research has shown that lacking satisfaction in terms of relatedness needs is negatively associated with emotional intelligence (58) and also impacts experienced emotions throughout the course of the day (12, 13). When specifically considering a lack of fulfillment of relatedness needs at work, there also appears to be a connection with affect. Loneliness at work has been shown to negatively predict affective organizational commitment (20, 26, 59), potentially because lonely employees are more detached from colleagues. As aforementioned, loneliness at work consists of two dimensions: emotional deprivation and a lack of social companionship (19). Increased feelings of insufficient social companionship at work are negatively correlated to employees' affective commitment (20). However, other research has indicated that both the emotional deprivation and social companionship facets of workplace loneliness have a negative impact on affective organizational commitment (59). Consequently, based on SDT and the aforementioned research results, we propose the following hypothesis:

*Hypothesis 1: Loneliness at work will negatively predict affective organizational commitment*.

### Workplace Loneliness and Relationships With Colleagues

Workplace relationships are interpersonal relationships characterized by continuous, patterned social interactions between individuals at work. Nearly all businesses require daily social interaction and strong workplace relationships in order to function properly (60). Relationships are especially important to consider in the context of the workplace, as they affect workplace attitudes and performance. For instance, quality leader-subordinate relationships have been shown to promote information retention and protect against turnover intention (61, 62). Additionally, satisfaction with one's workplace relationships is negatively associated with individual strain, such as depression and frustration, at work (63).

There are two primary types of workplace relationships that have been shown to have critical implications for workplace functioning: those with peers and those with supervisors. Two common measures of these relationships are perceptions of coworker support (64) and supervisor support (65), respectively. In terms of coworker support, research has indicated that it is positively predictive of workplace creativity and negatively predictive of turnover intentions (66–68). Coworker support can also protect against the stressors of mistreatment by one's supervisor or organization, positively moderating the relationship between a reduction in work stress and job satisfaction (69). Studies have also shown that coworker support can serve to mollify the effects of emotional exhaustion that workers experience due to work overload [e.g., (66)]. Like coworker support, supervisor support has numerous work-relevant consequences. For example, supervisor support predicts creative work output (68, 70). Supervisor support also drives a number of additional work outcomes, including organizational commitment, employee performance, and job satisfaction, and turnover intentions (68, 71, 72).

From an SDT perspective, fulfillment of relatedness needs has been shown to be related to actual relationship quality (10, 12). In the specific context of the workplace, this connection between relatedness and relationships with coworkers also seems to hold true. Loneliness at work can be explained, in part, due to a dearth of high-quality relationships at work. In line with this notion, insufficient support from colleagues has been shown to be a strong contributor to a feeling of loneliness at work (19). Better-quality relationships with supervisors also lead to lower levels of loneliness at work (16, 73). Specifically, a lack of support from supervisors can result in feelings of suspicion and fear that serve to promote workplace loneliness (73). Other research has demonstrated that certain leadership styles, such as paternal approaches to leadership, are associated with decreased levels of workplace loneliness (74). Overall, the research trends point to a negative relationship between loneliness at work and both supervisor and coworker support. Accordingly, we posit the following:

*Hypothesis 2: Loneliness at work will negatively predict a) coworker and b) supervisor support*.

### Workplace Loneliness and Behavioral Outcomes

Employee behavior has the potential to have a measurable impact on outcomes at the organization level. Two types of behavior that have been shown to be particularly influential are task and contextual performance. First, task performance is conceptualized as an individual's productivity at work, comprising both the accuracy and efficiency with which individuals carry out assigned tasks (75). Workplace performance is important to understand because it can be impacted by a multitude of variables including, but not limited to, the physical environment, supervisor support, coworker support, and loneliness at work (8, 16, 76). On the other hand, contextual performance—also known as organizational citizenship behaviors (OCBs)—constitute any contributions to the organization or work community that are not required of employees (77). OCBs are necessary to any workplace or business because of the potential consequences these behaviors have on performance; research has shown that higher amounts of organizational citizenship behaviors often result in increased performance volume and quality (78).

From the literature on SDT, a lack of relatedness can lead to alterations in individuals' behavior (11). This pattern also appears to hold true in the workplace, as loneliness at work has been shown to negatively correlate with job performance (8, 79). Self-evaluations as well as peer and supervisor evaluations have revealed those who are lonelier at work to have lower performance ratings (80). Loneliness at work may also indirectly predict worse job performance by mediating the relation between work alienation and job performance (81). Results of similar research have suggested that loneliness at work leads to reduced job satisfaction and, consequently, decreased performance (21). Although the authors are not aware of any research connecting workplace loneliness and organizational citizenship behaviors, we expect that a similar pattern will emerge. Accordingly, we posit the following hypothesis:

*Hypothesis 3: Loneliness at work will negatively predict a) organizational citizenship behaviors and b) perceived performance*.

## Working From Home as a Moderator

One of the benefits of working from home is increased worker autonomy (82); some scholars even refer to working from home as *locational autonomy* [e.g., (83)]. Through the lens of SDT (9), working from home exemplifies autonomy, which is another theorized area of psychological need.

Extant research suggests that autonomy brings about positive, affective responses in people of all ages (84). Autonomy has also been shown to positively correlate with social functioning (85). Finally, autonomy has been shown to positively impact behavioral outcomes; for instance, autonomy has been linked to the enactment of prosocial behaviors (86).

Accordingly, we believe that autonomy will moderate the relation between workplace loneliness and work-relevant outcomes. Although a dearth of fulfillment of relatedness needs is expected to have a deleterious impact on emotions, relationships, and behaviors, it also stands to reason that fulfillment of autonomy needs may mitigate that harmful effect. Thus, we expect that working from home (autonomy) will mollify the relationship between workplace loneliness (lack of relatedness) and affective, relational, and behavioral work outcomes. As such, we posit the following hypothesis:

*Hypothesis 4: Working from home will moderate the relation between workplace loneliness and a) affective, b) relational, and c) behavioral outcomes, such that the deleterious impact of loneliness on outcomes will be less pronounced for individuals who work from home*.

**Figure 1:**
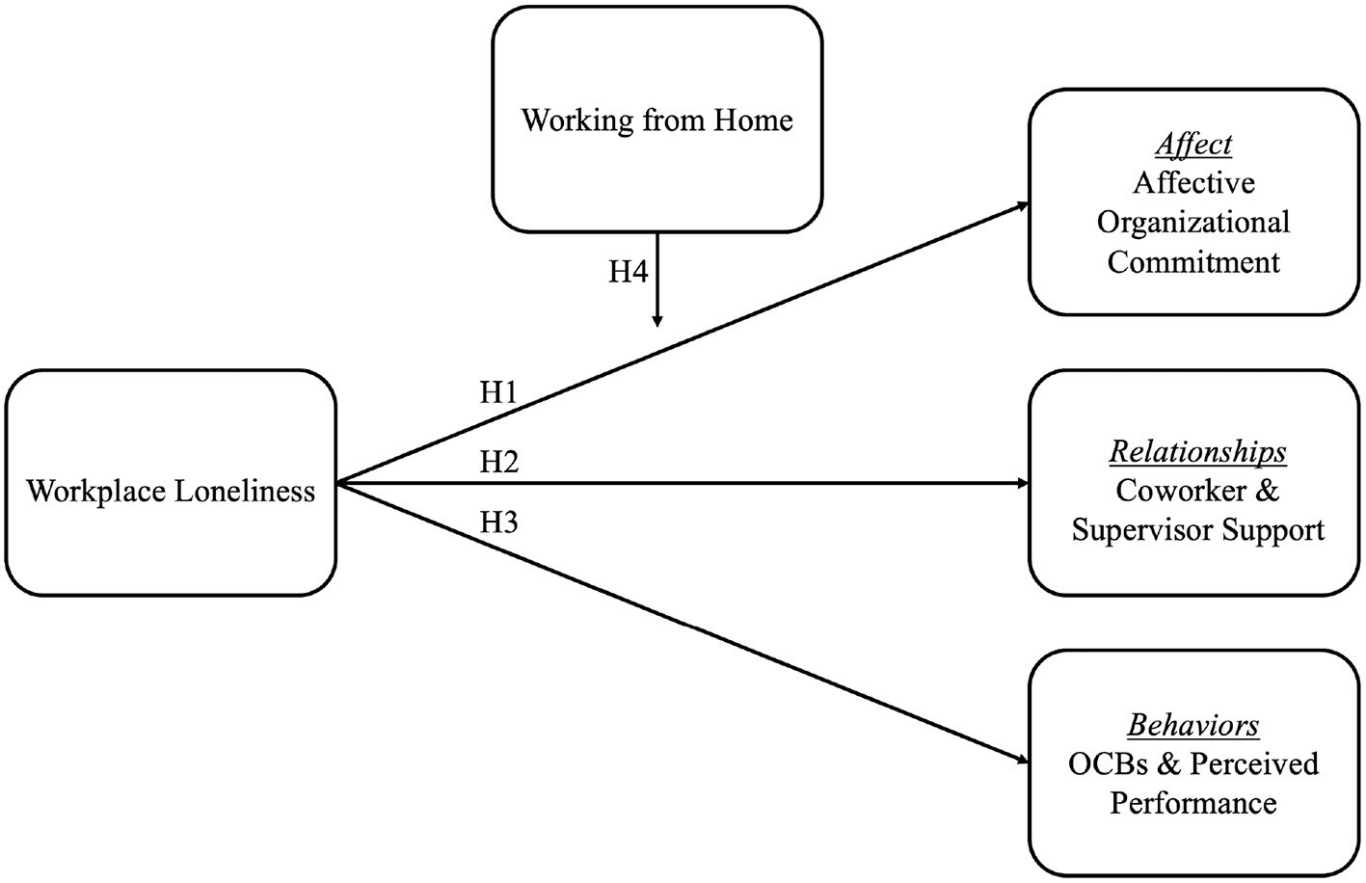
Theoretical model.

For a visual rendition of our full theoretical model, please see Figure 1.

## Methods

The current study employed a quantitative, survey-based, cross-sectional methodology, the details of which are described below. All data were collected using Qualtrics (87), a software survey system.

### Participants

Five-hundred and sixty-one survey responses were received. Of these responses, 391 completed surveys were usable for analyses. Responses that were eliminated from the final sample included: individuals who didn't respond to any questions (*n* = 92), individuals who were unemployed and had never previously been employed (*n* = 51), individuals who did not provide consent for their data to be used (*n* = 15), responses that came from the research group testing out the survey (*n* = 11), and one response that, based on respondent location and pattern of answers, appeared to be a duplicate response (*n* = 1). Of the 391 usable data points, 100% of individuals were currently employed or had previously been employed. Participants reported working in a variety of industries, including education (18.93%), dining (11.25%), sales (9.21%), healthcare (9.21%), business (7.93%), and administration (6.39%). The average age of the sample was 32.41 years (*SD* = 15.90). Please see Table 1 for additional information on participant demographics.

**Table 1:** Participant demographics.

**Demographic category**	**Percent of sample**
Working from home	
Yes	50.64
No	47.31
No response	2.05
Normally work from home?
All of the time	4.35
Some of the time	16.62
Never	74.68
No response	4.35
Laid off/furloughed due to COVID-19	
Yes	17.65
No	80.31
No response	2.05
Essential worker	
Yes	29.67
No	68.03
No response	2.30
Career stage	
Early	60.87
Mid	17.39
Late	20.20
No response	1.53
Salaried or hourly	
Salaried	26.60
Hourly	53.20
Different at different jobs	16.62
No response	3.58
Race/ethnicity	
American Indian or Alaska Native	1.02
Asian	9.97
Black or African American	7.93
Hispanic or Latinx	8.18
Middle Eastern	1.53
Native Hawaiian or Pacific Islander	0.26
White	61.13
Other	8.18
No response	1.79
Primary language	
English	94.12
Spanish	1.79
Other	1.79
No response	2.30
Country of birth	
USA	89.00
Other	8.18
No response	2.81
Gender	
Male	30.18
Female	66.24
Non-binary/third gender	1.02
Prefer to self-describe	0.51
Prefer not to say	0.26
No response	1.79
Transgender	
Yes	1.28
No	96.16
No response	2.56
Sexual orientation	
Straight/heterosexual	74.68
Gay or lesbian	5.12
Bisexual	11.25
Prefer to self-describe	3.84
Prefer not to say	2.81
No response	2.30
Religion	
Christianity (not including catholicism)	26.09
Catholicism	14.07
Judaism	9.97
Islam	1.02
Buddhism	1.53
Hinduism	0.51
Atheist	15.86
Agnostic	19.95
Other	7.16
No response	3.84
Political Party	
Democratic	69.82
Republican	9.72
Other	16.88
No response	3.58
Highest degree	
High school degree or equivalent	23.27
Some college, no degree	28.13
Associate degree	4.60
Bachelor's degree	23.02
Master's degree	12.79
Professional degree	3.07
Doctorate	3.32
No response	1.79
Marital status	
Single	64.19
Married	28.64
Separated	0.77
Divorced	3.84
Widowed	0.51
No response	2.05

*Categories that were not selected by any participants were removed from the table. Numbers have been rounded to the second decimal place*.

### Procedure

The population of interest in this study was employed, American adults. In order to derive a sample from that population, participants were primarily recruited to complete a web-based survey from a database of U.S. job candidates from a talent acquisition and development consulting company, but also partially from an alumni network from a small, liberal arts college in Southern California and social media posts. From the database of U.S. job candidates, 33,140 candidates were contacted *via* a personalized email with a link to the survey. These individuals were selected based on the following criteria: a) residing in the United States, b) having updated their candidate record within the past 5 years, and c) having a valid email address. The job candidates in the database include individuals who work in a variety of industries such as manufacturing, finance, health, pharmaceuticals, consumer products, IT, and engineering. These candidates range from no work experience to those who are mid- and late-career professionals. This database includes individuals in both trade as well as professional occupations.

### Measures

The following measures were used to capture conceptual variables in the current study. Survey items were not adapted or modified in any way.

#### Workplace Loneliness

Workplace loneliness was measured using Wright et al. (88) 16-item scale. An example item is as follows: “I often feel isolated when I am with my coworkers.” Response options ranged from “strongly disagree” (1) to “strongly agree” (7). Cronbach's alpha for this scale was 0.93.

#### Affective Organizational Commitment

Affective organizational commitment was captured using Allen and Meyer's (54) eight-item scale. An example item is as follows: “I would be very happy to spend the rest of my career with this organization.” Response options ranged from “strongly disagree” (1) to “strongly agree” (7). Cronbach's alpha for this scale was 0.86.

#### Coworker and Supervisor Support

Perceptions of coworker support were captured using O'Driscoll et al. (64) four-item scale. An example item is as follows: “How often, over the past 3 months, have you received sympathetic understanding and concern from colleagues?” Response options ranged from “never” (1) to “all the time” (6). Cronbach's alpha for this scale was 0.92.

Perceptions of supervisor support were captured using Kottke and Sharafinski (65) 16-item scale. An example item is as follows: “My supervisor really cares about my well-being.” Response options ranged from “strongly disagree” (1) to “strongly agree” (7). Cronbach's alpha for this scale was 0.96.

#### Organizational Citizenship Behaviors

OCBs were measured using Smith et al. (77) 16-item scale. Participants were instructed to respond about their own behavior. An example item is as follows: “Makes innovative suggestions to improve department.” Response options ranged from “very uncharacteristic” (1) to “very characteristic” (5). Cronbach's alpha for this scale was 0.79.

#### Job Performance

Perceived job performance was measured using Bal and De Lange (89) 3-item scale. An example item is as follows: “How would you rate your job performance, as an individual employee?” Response options ranged from “very poor” (1) to “excellent” (5). Cronbach's alpha for this scale was 0.84.

#### Demographics

Work-related and social demographics were measured by asking participants to self-report their demographic categories. Please see Table 1 for detailed information on the demographic items that participants responded to. In addition to the items listed in Table 1, we also asked participants to self-report their age, current employment status, and employment industry.

#### Covariates

General loneliness, which was included in analyses as a covariate, was measured using Rubenstein and Shaver (90) eight-item scale. An example item is as follows: “I am a lonely person.” All questions utilized Likert-type response formats, although the number of response options and scale points varied from question to question. Cronbach's alpha for this scale was 0.88.

## Results

All measured variables were deemed to be normally distributed. Descriptive statistics and Pearson's correlations were calculated for all of the study's primary variables, in addition to alphas coefficients, which were all deemed to be acceptable based on the standard academic interpretation of this statistic (91). These results can be seen in Table 2. Notably, loneliness at work and working from home were not significantly correlated with one another.

**Table 2:** Descriptive statistics and bivariate correlations.

**Variable**	***N***	***M***	***SD***	**1.**	**2.**	**3.**	**4.**	**5.**	**6.**	**7.**	**8.**
1. Work from home^+^	383	0.52	0.50	—							
2. General loneliness	391	2.23	0.68	−0.10	(0.88)						
3. Workplace loneliness	391	2.93	1.09	−0.06	0.34^***^	(0.93)					
4. Affective commitment	387	4.20	1.33	0.19^***^	−0.18^***^	−0.52^***^	(0.86)				
5. Coworker support	386	3.81	1.20	0.13^*^	−0.16^**^	−0.56^***^	0.49^***^	(0.92)			
6. Supervisor support	371	3.95	0.86	0.19^***^	−0.22^***^	−0.49^***^	0.53^***^	0.53^***^	(0.96)		
7. OCBs	390	3.90	0.54	−0.05	−0.21^***^	−0.29^***^	0.28^***^	0.26^***^	0.26^***^	(0.79)	
8. Perceived job performance	387	4.36	0.60	0.00	−0.31^***^	−0.29^***^	0.23^***^	0.29^***^	0.28^***^	0.51^***^	(0.84)

* *p < 0.05*.

** *p < 0.01*.

*** *p < 0.001*.

*Cronbach's alphas appear in parentheses along the diagonal.*

+ *This variable was coded as follows: 0 = no; 1 = yes*.

Next, in order to test Hypotheses 1 through 4, a single path analysis was run. General loneliness was included as a control variable, in order to ensure that the pattern of results was driven by workplace loneliness, specifically. Detailed results of this path analysis can be found in Table 3.

**Table 3:** Path analysis regressing affective organizational commitment, coworker support, supervisor support, OCBs, and perceived job performance on workplace loneliness, working from home, and their interaction (*N* = 362).

**Predictors**	**Affective organizational commitment**				**Coworker support**			
	***R*^2^ = 0.31**				***R*^2^ = 0.31**			
	**Estimate**	***SE***	***z*-value**	***p*-value**	**Estimate**	***SE***	***z*-value**	***p*-value**
General loneliness^*^	0.00	0.09	0.03	0.979	0.08	0.08	0.92	0.358
Workplace loneliness (WL)	−0.74	0.08	−9.57	0.000	−0.70	0.07	−10.01	0.000
Work from home (WFH)^+^	−0.29	0.33	−0.87	0.383	−0.33	0.30	−1.12	0.265
WL × WFH	0.24	0.11	2.29	0.022	0.20	0.09	2.09	0.036
**Predictors**	**Supervisor support**				**OCBs**			
	***R*^2^ = 0.29**				***R*^2^ = 0.12**			
	**Estimate**	***SE***	***z*-value**	***p*-value**	**Estimate**	***SE***	***z*-value**	***p*-value**
General loneliness^*^	−0.06	0.06	−1.00	0.319	−0.10	0.04	−2.39	0.017
Workplace loneliness (WL)	−0.41	0.05	−8.13	0.000	−0.19	0.04	−5.27	0.000
Work from home (WFH)^+^	0.02	0.21	0.11	0.912	−0.43	0.15	−2.86	0.004
WL × WFH	0.09	0.07	1.25	0.211	0.12	0.05	2.41	0.016
**Predictors**	**Perceived job performance**							
	***R*^2^ = 0.13**							
	**Estimate**	***SE***	***z*-value**	***p*-value**	
General loneliness^*^	−0.21	0.05	−4.54	0.000				
Workplace loneliness (WL)	−0.13	0.04	−3.36	0.001				
Work from home (WFH)^+^	−0.19	0.17	−1.10	0.271				
WL × WFH	0.05	0.05	0.87	0.385				

*SE, standard error. Estimates are standardized. CFI = 1.00. TLI = 1.00. RMESA = 0.00; 90% CI = 0.00, 0.00. SRMR = 0.00. χ^2^ = 693.39, df = 30, p < 0.001.*

* *Included as a covariate*.

+ *Coded as follows: 0 = no; 1 = yes*.

First, in order to test Hypothesis 1—which posited that loneliness at work would negatively predict affective organizational commitment—affective organizational commitment was regressed onto loneliness at work. Results suggested that loneliness at work negatively predicts affective organizational commitment (estimate = −0.74, *p* < 0.001). Accordingly, Hypothesis 1 was supported.

Second, in order to test Hypothesis 2a—which posited that loneliness at work would negatively predict coworker support—coworker support was regressed onto loneliness at work. Results suggested that loneliness at work negatively predicts coworker support (estimate = −0.70, *p* < 0.001). Thus, Hypothesis 2a was supported.

Third, in order to test Hypothesis 2b—which posited that loneliness at work would negatively predict supervisor support—supervisor support was regressed onto loneliness at work. Results suggested that loneliness at work negatively predicts supervisor support (estimate = −0.41, *p* < 0.001). Accordingly, Hypothesis 2b was supported.

Fourth, in order to test Hypothesis 3a—which posited that loneliness at work would negatively predict OCBs—OCBs were regressed onto loneliness at work. Results suggested that loneliness at work negatively predicts OCBs (estimate = −0.19, *p* < 0.001). Thus, Hypothesis 3a was supported.

Fifth, in order to test Hypothesis 3b—which posited that loneliness at work would negatively predict perceived performance—perceived performance was regressed onto loneliness at work. Results suggested that loneliness at work negatively predicts perceived performance (estimate = −0.13, *p* < 0.01). Accordingly, Hypothesis 3b was supported.

**Figure 2:**
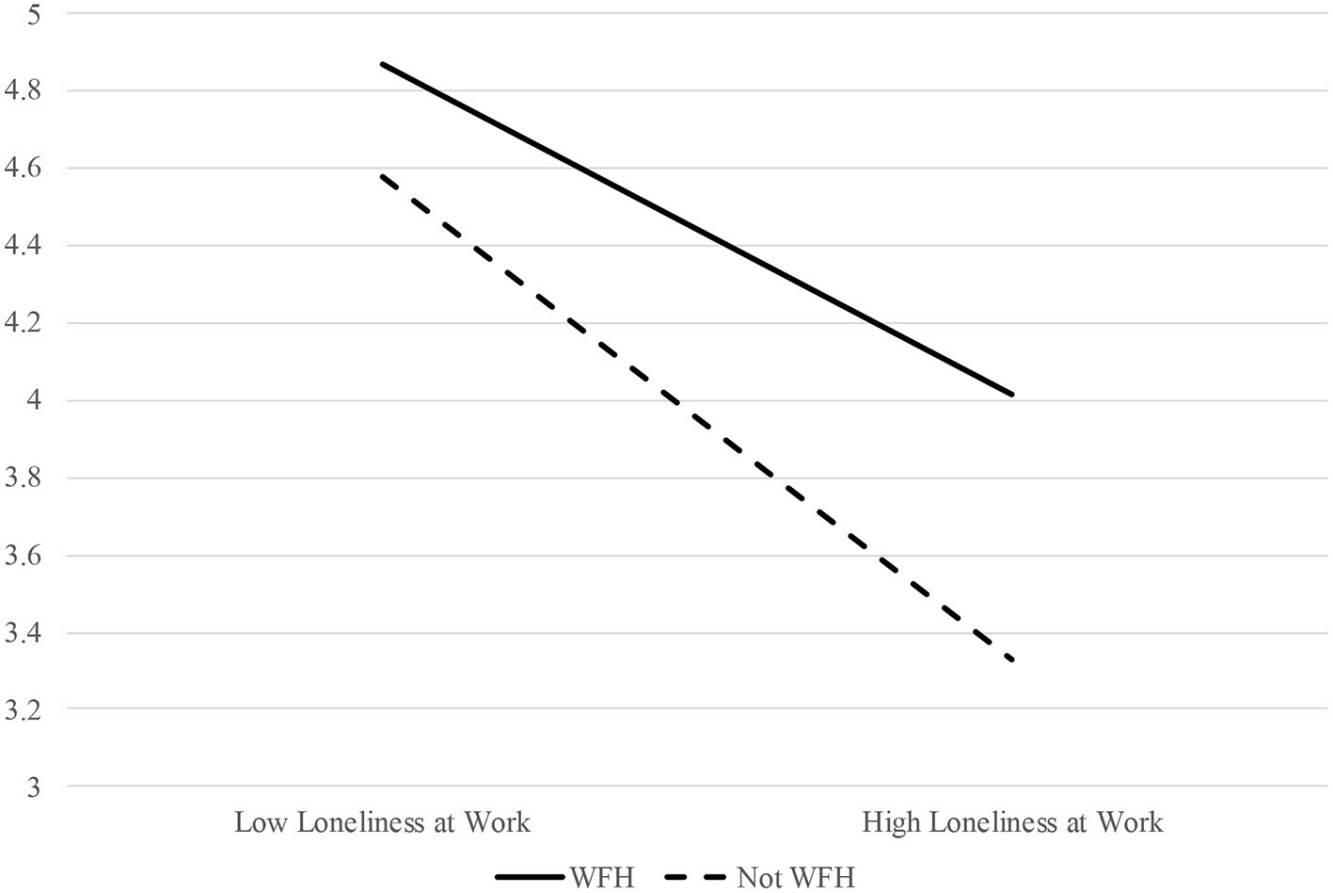
The interactive effect of workplace loneliness and working from home (WFH) on affective organizational commitment.

Sixth, in order to test Hypothesis 4a—which posited that working from home would moderate the relation between workplace loneliness and affective outcomes, such that the deleterious impact of loneliness on affective outcomes will be less pronounced for individuals who work from home—the interactive effect of workplace loneliness and working from home was tested as a predictor of affective organizational commitment. Results suggested that loneliness at work and working from home have an interactive effect on affective organizational commitment in the expected direction (estimate = 0.24, *p* < 0.05). Accordingly, Hypothesis 4a was supported. For a visualization of the interactive effect of workplace loneliness and working from home on affective organizational commitment, please see Figure 2.

**Figure 3:**
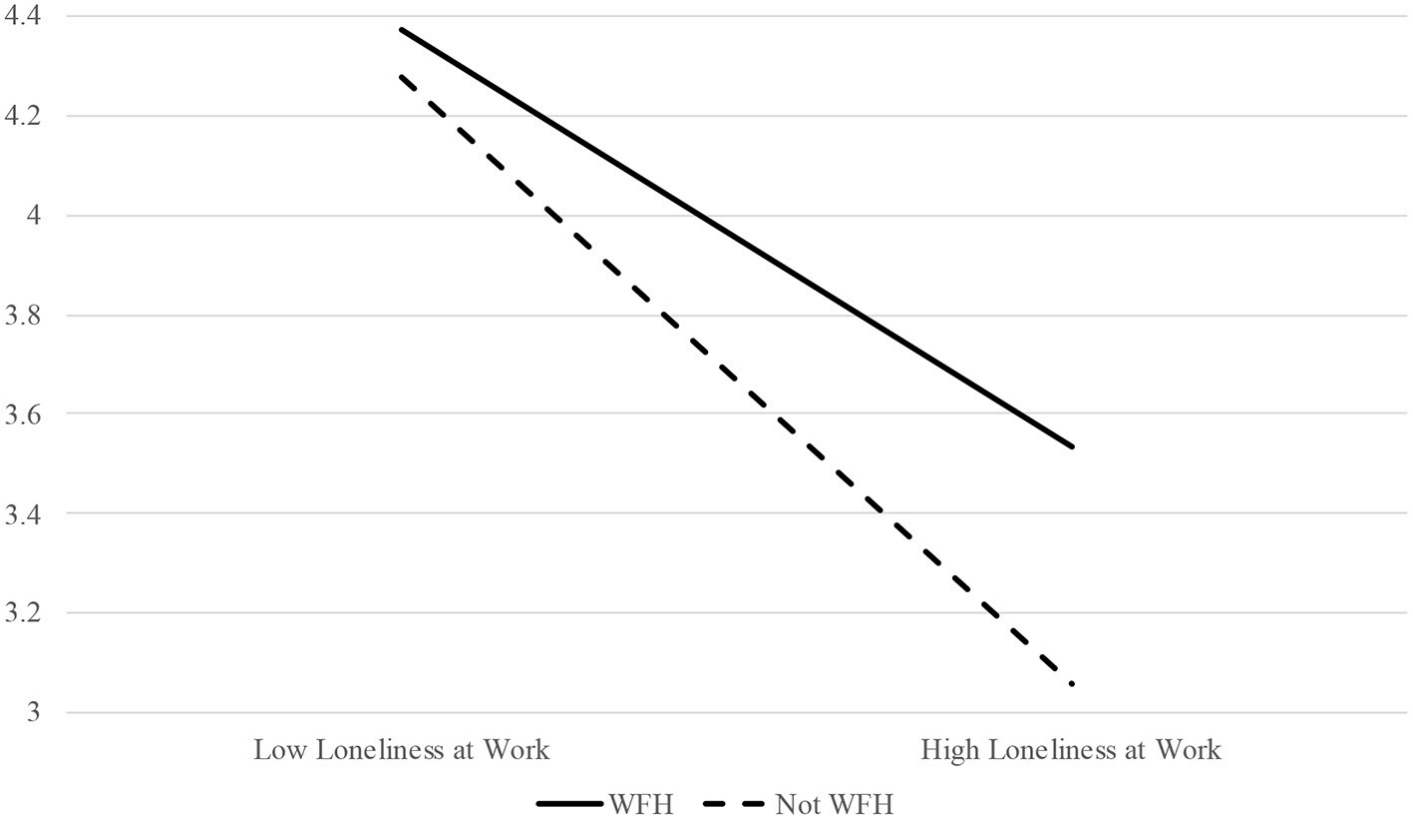
The interactive effect of workplace loneliness and working from home (WFH) on perceived coworker support.

Seventh, in order to test Hypothesis 4b—which posited that working from home would moderate the relation between workplace loneliness and relational outcomes, such that the deleterious impact of loneliness on relational outcomes will be less pronounced for individuals who work from home—the interactive effect of workplace loneliness and working from home was tested as a predictor of coworker and supervisor support. Results suggested that loneliness at work and working from home have an interactive effect on coworker support in the expected direction (estimate = 0.20, *p* < 0.05) but do not have an interactive effect on supervisor support (estimate = 0.09, *ns*). Accordingly, Hypothesis 4b was partially supported. For a visualization of the interactive effect of workplace loneliness and working from home on coworker support, please see Figure 3.

**Figure 4:**
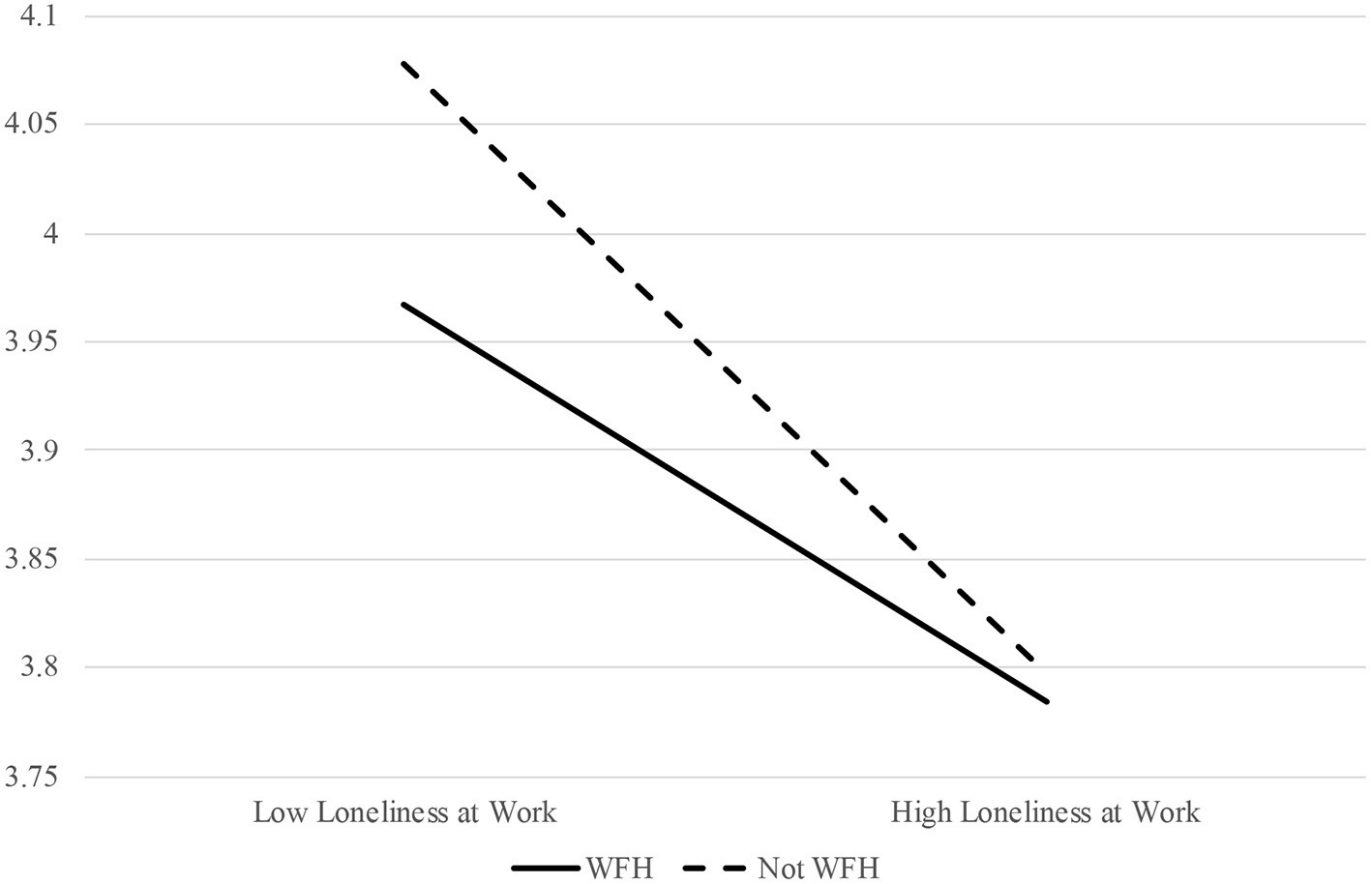
The interactive effect of workplace loneliness and working from home (WFH) on OCBs.

Eighth, in order to test Hypothesis 4c—which posited that working from home would moderate the relation between workplace loneliness and behavioral outcomes, such that the deleterious impact of loneliness on behavioral outcomes will be less pronounced for individuals who work from home—the interactive effect of workplace loneliness and working from home was tested as a predictor of OCBs and perceived performance. Results suggested that loneliness at work and working from home have an interactive effect on OCBs, but not in the expected direction (estimate = 0.12, *p* < 0.05). However, loneliness at work and working from home did not have an interactive effect on perceived job performance (estimate = 0.05, *ns*). Accordingly, Hypothesis 4c was not supported. For a visualization of the interactive effect of workplace loneliness and working from home on OCBs, please see Figure 4.

## Discussion

The current study explored the interactive effect of workplace loneliness and working from home on a number of work-relevant outcomes, through the theoretical lens of SDT (9). Overall, results suggested that a dearth of relatedness (i.e., workplace loneliness) negatively predicted affective organizational commitment, perceptions of coworker and supervisor support, organizational citizenship behaviors, and perceived performance. Moreover, autonomy (i.e., working from home) partially mitigated the negative effect that workplace loneliness has on perceptions of coworker and supervisor support. Autonomy also was found to buffer the negative relation between lack of relatedness and affective organizational commitment, but it exacerbated the negative relation between lack of relatedness and OCBs. A detailed discussion of these results can be found below.

### Summary of Results

Providing support for Hypotheses 1 through 3, our results indicated that loneliness at work negatively predicts affective organizational commitment, perceptions of coworker support, perceptions of supervisor support, OCBs, and perceived performance. In support of Hypothesis 4a, results suggested that working from home moderates the relation between workplace loneliness and affective organizational commitment, such that working from home weakens the deleterious impact of workplace loneliness on affective organizational commitment. In partial support of Hypothesis 4b, results suggested that working from home moderates the relation between workplace loneliness and perceptions of coworker support, such that working from home weakens the deleterious impact of workplace loneliness on perceptions of coworker support. However, working from home did not have a significant moderating impact on the relation between workplace loneliness and perceptions of supervisor support, as was expected. Finally, results suggested that loneliness at work and working from home have an interactive effect on OCBs, but not in the expected direction; working from home strengthened the deleterious impact of workplace loneliness on OCBs. In terms of perceived performance, our results suggested that there was not a significant interactive effect of workplace loneliness and working from home. Thus, Hypothesis 4c was not supported.

### Theoretical Contributions

First, the current paper presents a novel application of SDT (9). While SDT has been demonstrated as an effective theory of motivation in many contexts [e.g., (10–13)], the specific experience of working from home has yet to be thoroughly explored through the lens of SDT. The current paper applied SDT to this context, providing a framework for future researchers to extent this line of thinking in the future.

Second, in terms of direct effects of workplace loneliness, our results suggest a negative relation to affective, relational, and behavioral outcomes. First, workplace loneliness was shown to negatively predict affective organizational commitment, as was hypothesized. This implies that loneliness at work has a measurable impact on the way that employees feel about their employer; less lonely employees feel more connected and committed to their employer, and vice versa. Second, workplace loneliness negatively predicted perceptions of both coworker and supervisor support. This finding reaffirms extant literature on the linkage between coworker relationships and loneliness, which has also shown that the two are negatively correlated with one another (19). Finally, workplace loneliness negatively predicted both of the behaviors that were measured in this study: organizational citizenship behaviors and perceived performance. The negative relation between loneliness at work and perceived performance is of particular interest, since perceived performance can be thought of as an operationalization of competence, the third type of psychological need delineated by SDT (9). So, our results also support the notion that relatedness and competence are interrelated needs, a finding that has been demonstrated in previous literature [e.g., (12)].

Finally, in line with our hypotheses, workplace loneliness and working from home had an interactive effect on both affective organizational commitment and perceptions of coworker support, such that very lonely people were more committed to their organizations and perceived their coworkers as more supportive when they worked from home as opposed to not. So, in line with SDT (9), it seems that fulfillment of autonomy needs buffers the negative impact that lack of fulfillment of relatedness has on affective and relational work outcomes.

Contrary to our hypothesis, however, workplace loneliness and working from home had an interactive effect on OCBs such that people who *did not* work from home tended to engage in more OCBs than people who did, and this was especially true for people who were less lonely. This result suggests that working from home takes a toll on the ability of workers to enact OCBs. Logistically, this finding makes sense; many OCBs hinge on in-person interaction, such as noticing that a colleague needs help and pitching in to assist. In support of this notion, research from the era of the COVID-19 pandemic has indicated that employees are seemingly less prone to engaging in behaviors that emerge most readily in face-to-face contexts; for instance, collective action (92).

Finally, workplace loneliness and working from home did not have an interactive effect on perceptions of supervisor support or perceived performance. So, while our results suggest that lack of relatedness (workplace loneliness) and autonomy (working from home) have an interactive impact on certain affective, relational, and behavioral work-relevant outcomes, these interactive impacts did not apply to all variables that we measured in the current study. One possible explanation for this pattern of results is that perceptions of supervisor support and perceived performance are particularly resilient to the impact of working from home. In other words, regardless of whether individuals are working from the office or home, the relation between workplace loneliness and perceptions of supervisor support/perceived performance are similar, implying that the impact that loneliness has on these outcomes may not be mitigated by the location where work takes place.

### Limitations

Our study has several limitations that we would like to broach. First, the current study is cross-sectional in nature, which limits our ability to make causal inferences. In fact, we urge readers not to draw causal inferences of any kind based off of the current study, due to the fact that our design is not conducive to inferring cause and effect. Second, our study is subject to the mono-method bias. Because our results are entirely based off of self-report, survey-based data, it is possible that effect sizes have been artificially aggrandized.

Third, our sample may have issues of generalizability. As seen in Table 1, while our sample was diverse in many ways, it was not entirely reflective of the larger U.S. population of working adults. So, these results may not generalize to all people in all places. Fourth, perceived job performance is a relatively weak measure of performance, since it is subject to the social desirability bias. In other words, because performance was self-reported, results related to performance may not be accurate.

Finally, the current study did not measure working from home as a continuous variable, and also did not capture degree of interaction with colleagues whilst working from home. Both of these metrics would be an interesting direction for future researchers to capture, in terms of their operationalization of working from home and assessment of covariates.

### Future Directions

In terms of future directions that are based off of the current study's limitations, we recommend that future researchers endeavor to study the interactive effect of workplace loneliness and working from home on work-relevant outcomes using longitudinal, multi-methodological research designs with large, diverse samples of workers. We also recommend that, for future studies that focus on performance as an outcome, researchers collect relatively objective measures of performance.

In addition, future researchers should venture to explore whether the experiences of people who work from home are fundamentally different during a global pandemic vs. not. Is the COVID-19 pandemic driving the current study's results, either fully or in part? Moving forward, collecting data for purposes of comparison, during and after the pandemic, would help to shed light on this question. Furthermore, we suggest that future researchers explore working from home as a continuous rather than a categorical variable. In reality, working from home is a spectrum; thus, one avenue for future work would be to operationalize this variable as such, and glean results accordingly.

We also suggest that future researchers investigate cross-compare experiences of workplace loneliness in virtual organizations (i.e., organizations where all employees work remotely) as compared with traditional organizations. Does it matter where the majority of workers work from? With many large employers suggesting that half of their workforce may continue to work from home post-pandemic, researchers will have unique opportunities to explore the dynamics of organization, work, and job design on workplace loneliness and its effect on workplace outcomes.

### Applied Implications

With statistically significant negative correlations between loneliness at work and affective organizational commitment, coworker support, supervisor support, OCBs, and perceived job performance (see Table 2), strategies to minimize loneliness at work are strongly recommended, as employers who mitigate workplace loneliness will potentially reduce employee turnover and increase OCBs and job performance. The current study found that less lonely employees have higher levels of affective organizational commitment, signifying that tackling workplace loneliness will reduce employee turnover and the costs of recruiting and training replacements.

Accordingly, we recommend that employers strategically nurture positive, supportive relationships among coworkers and supervisors, as this may reduce loneliness in the workplace. This can be done by facilitating relationship-building amongst employees *via* interventions such as team building, networking events, or structured social hours. While it may seem that employees spending time at work focusing on activities that are not directly work-relevant would hurt organizational productivity, the results of the current study demonstrate just the opposite; less lonely employees end up with the best outcomes for themselves and the organization as a whole, and these employees are the same people who likely carved out time at work to develop high-quality relationships. In creating opportunities for relationships between coworkers to develop, employers should focus on the quality of the relationships that employees build with one other rather than the number of relationships. Strong, supportive, high-quality relationships with coworkers and supervisors are the bonds that mitigate loneliness.

The current study also found that loneliness at work is not tied to where a person works; employees working from home were just as lonely as those working from corporate offices. However, those working from home reported higher affective organizational commitment and coworker/supervisor support levels than those who did not, suggesting that it may be better to be lonely working from home than from a corporate office. That being said, we caution the reader not to overgeneralize this observation. The COVID-19 pandemic created unique work-from-home circumstances where employees had to redesign their jobs and workflows in order to do from home what they once did from corporate offices. With many people working exclusively from home during the pandemic, coworkers may be more likely to initiate frequent calls and web-based meetings with each other to get work done. Consequently, these unique circumstances may have increased coworker interactions, perceived levels of coworker and supervisor support, and lowered feelings of loneliness. Overall, our results suggest that workplace loneliness is not dependent on where an employee works but rather on the quality of their relationships with peers and supervisors.

Finally, employers need to facilitate ways for employees to make OCB-type contributions to the workplace from home. The current study found that the negative relation between workplace loneliness and OCBs was exacerbated for individuals who worked from home. This is likely because OCBs tend to be contingent on coworkers observing peers in need of help. When the workplace is the home, peers and supervisors are not as easily observable as they would be in an office. Thus, employers should consider training programs to teach managers how to help work-from-home employees contribute OCB-type behaviors, in addition to training programs for work-from-home employees to assist them with contributing OCB-type behaviors to the same extent as they would if working from a corporate office.

## Conclusion

Through the lens of SDT (9), the current study sought to: a) clarify the direct impact of workplace loneliness on work-relevant outcomes, and b) unpack the potential moderating impact of working from home on the aforementioned relations between workplace loneliness and outcomes. Results suggested that workplace loneliness negatively predicts affective organizational commitment, perceptions of coworker and supervisor support, organizational citizenship behaviors, and perceived performance. Furthermore, results implied that workplace loneliness and working from home have an interactive effect on affective organizational commitment, perceptions of coworker support, and organizational citizenship behaviors. While working from home had a beneficial impact on the relation between workplace loneliness and affective/relational outcomes, it had a detrimental impact on the relation between workplace loneliness and behavioral outcomes. Our findings highlight the importance of facilitating opportunities for employees to engage in OCBs from home.

## Data Availability Statement

The raw data supporting the conclusions of this article will be made available by the authors, without undue reservation.

## Ethics Statement

This study, which involved human participants, was reviewed and approved by Occidental College's IRB. Participants provided their written informed consent to participate in this study.

## Author Contributions

All authors listed have made a substantial, direct, and intellectual contribution to the work and approved it for publication.

## Conflict of Interest

The authors declare that the research was conducted in the absence of any commercial or financial relationships that could be construed as a potential conflict of interest.

## Publisher's Note

All claims expressed in this article are solely those of the authors and do not necessarily represent those of their affiliated organizations, or those of the publisher, the editors and the reviewers. Any product that may be evaluated in this article, or claim that may be made by its manufacturer, is not guaranteed or endorsed by the publisher.

## Footnotes

^1^Working from home is also referred to as teleworking, remote working, and telecommuting. For the purposes of this paper, we will use these terms interchangeably.
